# Normative modeling reveals functional connectivity heterogeneity in internet gaming disorder

**DOI:** 10.1186/s12991-026-00664-3

**Published:** 2026-05-02

**Authors:** Zixiao Wang, Yong Xie, Yidan Wang, Lingxiao Wang, Guang-Heng Dong

**Affiliations:** 1 Centre for Cognition and Brain disorders / Department of Neurology, The Affiliated Hospital of Hangzhou Normal University, Hangzhou, Zhejiang Province China; 2 Institute of Psychological Science, Hangzhou Normal University, Hangzhou, Zhejiang Province China; 3 Zhejiang Key Laboratory for Research in Assessment of Cognitive Impairments, Hangzhou, Zhejiang Province China; 4 Shenzhen No.3 Vocational School of Technology, Shenzhen, 518038 Guangdong China; 5 Dept of Psychology, Shaanxi Provincial People’s Hospital, Xi’an, 710068 China; 6 Department of Psychology, Yunnan Normal University, Kunming, Yunnan Province China; 7 Centre for Cognition and Brain Disorders, Hangzhou Normal University, NO. 2318, Yuhangtang Road, Yuhang District, Hangzhou, 311121 Zhejiang Province China

**Keywords:** Internet gaming disorder, Functional connectivity, Heterogeneity, Normative modeling, Clustering analyses

## Abstract

**Background:**

Internet gaming disorder (IGD) is associated with abnormal functional connectivity (FC) in brain networks. However, findings from resting-state functional magnetic resonance imaging studies are highly inconsistent, likely due to individual heterogeneity in IGD-related neural alterations—a feature commonly observed in other psychiatric disorders but understudied in IGD.

**Methods:**

We applied normative modeling to nucleus accumbens (NAcc) seed-to-voxel resting-state FC to derive individualized deviation (Z) maps for 173 IGD participants relative to age- and sex-adjusted normative ranges from 232 healthy controls. We then performed exploratory unsupervised clustering of network-level deviation features across three sample data, three atlas templates, and two clustering algorithms, selecting the optimal number of clusters using the silhouette criterion.

**Results:**

IGD showed marked heterogeneity in FC deviations: voxel-level deviations were largely idiosyncratic in both spatial distribution and direction. When deviations were summarized at the network level, clustering consistently selected a two-cluster solution across data, atlases, and algorithms, separating a majority “low-deviation” stratum from a smaller “high-deviation” stratum.

**Conclusions:**

IGD is characterized by pronounced individual variability in FC deviations. Network-level deviations yield a robust higher- vs. lower-deviation stratification, although the present findings do not support interpreting this pattern as evidence for discrete subtypes. The present study highlights the utility of individualized deviation mapping beyond conventional case–control analyses for characterizing heterogeneity in IGD.

**Supplementary Information:**

The online version contains supplementary material available at 10.1186/s12991-026-00664-3.

## Background

Internet gaming disorder (IGD) refers to the problematic and excessive use of internet games, which can be detrimental to various aspects of one’s education and life [1, 2]. The American Psychiatric Association (APA) included IGD in Section III of the fifth edition of Diagnostic and Statistical Manual of Mental Disorders (DSM-5) as warranting more clinical research in 2013 [3]. Using resting-state functional magnetic resonance imaging (RS-fMRI), researchers have conducted numerous studies investigating the characteristics and underlying neural basis of IGD, suggesting some core neural abnormalities of IGD, such as abnormal brain functional networks related to cognitive control, reward processing [4–7].

However, after summarizing previous RS-fMRI studies on IGD, we found that it was difficult to draw a uniform conclusion about the underlying neural mechanism of IGD. For example, some researchers found that compared to healthy control (HC) group, IGD group displayed decreased functional connectivity (FC) between the dorsolateral prefrontal cortex (DLPFC) and nucleus accumbens (NAcc) [8], decreased FC between the orbitofrontal cortex (OFC) and putamen [9], and decreased FC between the anterior cingulate cortex and caudate [10]. On the other hand, some researchers identified increased FC between the OFC and putamen [11], increased FC between the DLPFC and posterior cingulate cortex [12], and increased FC between the middle frontal gyrus and caudate [13] in IGD group in comparison with HC group. Both the direction and the location of abnormalities in IGD group vary across RS-fMRI studies. Such inconsistency may arise from true neurobiological heterogeneity across individuals with IGD and/or from methodological variability across studies (e.g., sample characteristics, FC definitions, preprocessing, and analytic pipelines). We hypothesized that inter-individual heterogeneity of IGD in resting-state FC abnormalities may contribute substantially to these inconsistent results. Heterogeneity is a core feature of many disorders, including schizophrenia, autism spectrum disorder (ASD), bipolar disorder and substance addiction [14–18]. However, the heterogeneity of IGD is still poorly understood. If IGD is indeed highly heterogeneous, this would motivate approaches to characterize individual-level abnormality patterns and thus prompt more individualized treatment for IGD. However, group-level differences derived from comparisons between IGD and healthy groups in previous studies might not necessarily be representative of an individual IGD gamer. Such group-level effects may capture only a small fraction of the neural alterations associated with IGD. Moreover, these effects may be disproportionately driven by a subset of individuals who show large deviations from the normative range, as has been reported in other heterogeneous disorders, e.g., schizophrenia [14] and ASD [19].

Accordingly, the present study aimed to map the individualized abnormalities of brain FC in IGD using a recent methodology, normative modeling. Normative modeling has been well documented and applied in studies of heterogeneity in other psychiatric disorders, such as ASD, schizophrenia, depressive and bipolar disorder [14, 16, 20, 21]. A normative model can be understood as a statistical model that predicts the brain measurement and its distribution from the demographic variables such as age and sex. Using brain data from a large sample of healthy controls, we constructed normative models based on warped Bayesian linear regression (BLR) [22]. Within this normative-modeling framework, brain metrics are modeled as a function of covariates (e.g., age and sex), yielding the normative distribution (i.e., predictive mean and centiles) that describes variation of each brain metric in the healthy population given age and sex. In warped BLR, a likelihood-warping step applies a monotonic, invertible transformation to the brain metric so that the residuals are closer to Gaussian. Predictions and deviation scores are then transformed back to the original measurement scale for interpretation. Accordingly, warped BLR was chosen because it can handle non-Gaussian residuals through likelihood warping (which improves estimation of extreme centiles relevant for deviation inference), it supports principled Bayesian model selection via marginal likelihood, and it is computationally efficient and scalable for large-scale modeling [22]. We then compared each IGD participant’s brain metric with the corresponding age- and sex-specific normative prediction to quantify individualized deviations from the HC reference distribution. This constitutes the first goal of the present study. We expected that different IGD individuals would exhibit different deviations from normative models, i.e., reflecting heterogeneity in resting-state brain FC, as suggested by previous RS-fMRI studies.

Additionally, the bilateral NAcc was selected in the present study as the region of interest (ROI) for the seed-to-voxel resting-state FC analysis to compute the FC between the bilateral NAcc and the rest of the brain. The NAcc, the main projection region of the mesolimbic pathway, has been confirmed to be involved in driving craving and the development and maintenance of addiction across many addiction disorders, including IGD [23–25]. Moreover, many studies have revealed abnormal activation/FC of the NAcc in individuals with IGD [8, 26–28]. The second goal of the present study was to exploratorily characterize heterogeneity among individuals with IGD using clustering analyses. Clustering is a widely used data-driven approach for examining biological heterogeneity and for identifying potential patterns of stratification within a clinical cohort based on multivariate profiles of selected measures [20, 29]. Accordingly, we performed clustering analyses as an exploratory approach to examine whether individuals with IGD could be stratified according to the extent and direction of deviations from normative models of FC.

## Methods

### Participants

The present study included 173 university students with IGD (males/females: 98/75) and 232 HCs (males/females: 139/93). The sex distribution did not differ significantly between groups. All the participants were free of any psychiatric/neurological disorders confirmed by a structured psychiatric interview (Mini International Neuropsychiatric Interview). No participant reported illegal drug use, gambling, or excessive nicotine and alcohol use. The diagnosis of IGD was based on the Young’s Internet Addiction Test (IAT) [30] and DSM-5 IGD criteria [31]. Consistent with previous studies of IGD [4, 32–34], the diagnostic criteria for the IGD group included: (1) IAT score ≥ 50; (2) DSM-5 criteria score ≥ 5; and (3) spending most of their internet time playing online games. Participants who did not meet the IGD criteria (IAT ≥ 50 & DSM-5 ≥ 5) were classified as the HC group. The majority of HC participants did not exceed either of the diagnostic thresholds (i.e., IAT < 50 and DSM-5 < 5). A small number of HC participants exceeded one threshold but not the other. They may represent recreational game users with borderline characteristics. Importantly, they did not simultaneously meet al.l the criteria for IGD and can therefore serve as appropriate comparison participants. Accordingly, these individuals were retained in the HC group. Demographic and diagnostic characteristics are shown in Table 1.

**Table 1:** Demographic and diagnostic characteristics

Items	IGD	HC	χ^2^/t	*p*
Sex (Male/Female)	98/75	139/93	0.436	0.509
Age (years)	21.13 ± 2.33	21.53 ± 2.43	-1.663	0.097
IAT score	66.15 ± 9.13	40.32 ± 10.92	—	—
DSM-5 score	6.08 ± 1.13	2.51 ± 1.37	—	—

Table values are mean ± standard deviation. Sex was compared using a chi-squared test and age using an independent-samples t-test. IAT and DSM-5 scores were used for group classification and are therefore reported descriptively without inferential testing

IGD, Internet Gaming Disorder; HC, Healthy Control; IAT, Internet Addiction Test; DSM-5, the fifth edition of Diagnostic and Statistical Manual of Mental Disorders

### RS-fMRI data acquisition

All participants underwent a RS-fMRI scan for 7 min using a 3 T scanner (Siemens Trio, Malvern, PA, USA) equipped for echo-planar imaging (EPI). During the scan, they were asked to keep their eyes open and stay still and relaxed. The scan parameters were as follows: repetition time (TR) = 2000 ms, echo time (TE) = 30 ms, slice number = 33, interleaved sequence, slice thickness = 3 mm, voxel size = 3 × 3 × 3 mm^3^, field of view (FOV) = 220 × 220 mm^2^, flip angle = 90° and matrix of 64 × 64.

### Preprocessing of RS-fMRI data

Data preprocessing was performed using DPABI (Data Processing & Analysis for Brain Imaging) v8.2 [35], a MATLAB-based toolbox. The preprocessing steps included: (1) discarding the first 10 time points; (2) slice-timing; (3) realignment for head-motion correction; (4) spatial normalization to Montreal Neurological Institute 152 (MNI152) space; (5) spatial smoothing (FWHM = 6 mm); (6) linear trend removal; (7) nuisance covariates regression, including head-motion covariates using the Friston 24-parameter model as well as signals from white matter, cerebrospinal fluid, and global signals; (8) band-pass filtering with a range of 0.01–0.1 Hz.

### Constructing normative models of FC

**Fig. 1:**
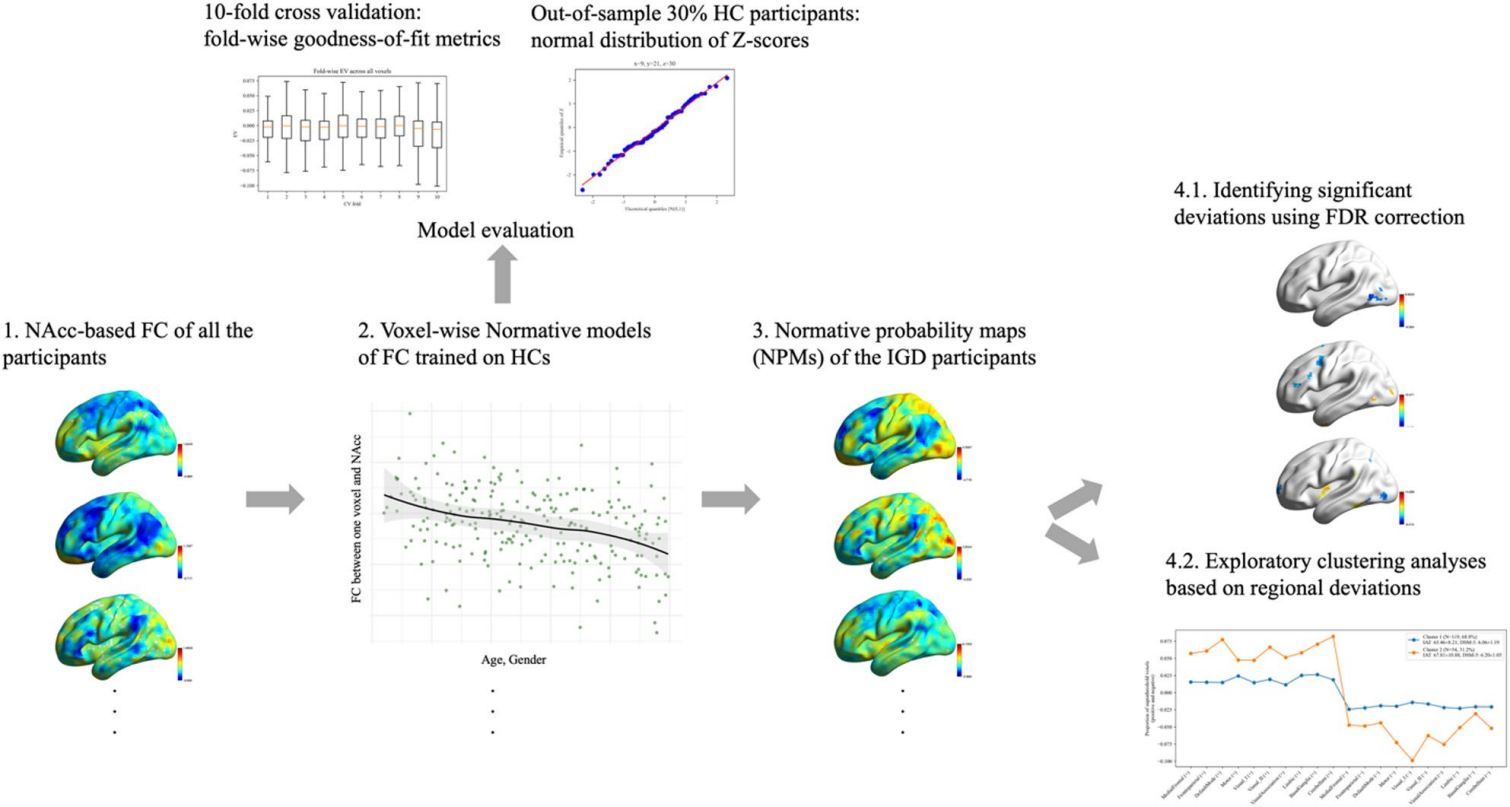
An overview of the normative modeling approach. IGD, Internet Gaming Disorder; HCs, Healthy Controls; NAcc, Nucleus Accumbens; FC, Functional Connectivity; FDR, False Discovery Rate

An overview of the normative modeling approach is provided in Fig. 1. First, seed-to-voxel FC maps were computed in DPABI v8.2 by calculating Pearson correlation coefficients (r) between the mean time series of the bilateral NAcc ROI and the time series of each voxel in the whole brain (i.e., voxel-wise correlations between the NAcc seed and every brain voxel). The resulting correlation maps were then Fisher r-to-z transformed prior to normative modeling. Second, we used warped BLR [22] implemented in PCNtoolkit (Predictive Clinical Neuroscience toolkit) [36] to model voxel-wise FC as a function of age and sex in the whole HC group (*N* = 232). To evaluate generalizability and guard against overfitting, we performed 10-fold cross-validation (CV) within the HC group. In each fold, 90% of the HC participants were used to train the normative model and the remaining 10% served as a held-out test set, using 10 non-overlapping folds such that each participant appeared in the test set exactly once. For each fold, the trained voxel-wise normative model produced both the predicted mean FC and predictive uncertainty at each voxel. Then, the trained model was used to place each test participant from 10% of the HC participants within the normative range and then quantify their deviations (i.e., Z scores) of FC from the healthy range at each specific brain voxel. Specifically, for participant *n* at voxel *d*, the Z score was calculated as:

$$\:{Z}_{nd}=\frac{({y}_{nd}-{\widehat{y}}_{nd})}{\sqrt{{{\sigma\:}_{d}}^{2}+{{(\sigma\:}_{*}^{2})}_{d}}}$$

where $\:{y}_{nd}$ is the observed value and $\:{\widehat{y}}_{nd}$ is the model-predicted mean. The denominator combines the estimated noise variance $\:{{\sigma\:}_{d}}^{2}$ (i.e., residual variance) and the predictive variance due to modeling uncertainty $\:{{(\sigma\:}_{*}^{2})}_{d}$, following the warped BLR normative-modeling formulation described in [22]. Using this equation, a normative probability map (NPM; i.e., a voxel-wise Z-score map) was generated for each test participant. After the 10-fold CV, all the HC participants had their corresponding NPMs derived from the fold in which they were in the test set. For each fold and each voxel, the goodness-of-fit metrics, including the explained variance (EV) and the mean standardized log loss (MSLL), were also yielded. The fold-wise EV and MSLL across all voxels are summarized (median and interquartile range) in Supplementary Figure S1, showing similar and stable model performance across folds, suggesting no clear evidence of severe overfitting.

Moreover, referring to [37], beyond fit metrics (e.g., EV and MSLL), validating whether the Z-scores of the test set follow a normal distribution is essential when evaluating model performance. To do so, we randomly split the HC participants into a 70% “normative training” subset (*N* = 162) and a 30% “normative test” subset (*N* = 70), stratified by age and sex. The voxel-wise normative model was fitted on the 70% training subset, and then used to generate the NPM (i.e., voxel-wise Z-score map) for each of the held-out 30% HC participants. For each voxel, we calculated the mean, variance, skewness, and kurtosis of Z-scores across the held-out 30% HC participants. The median and interquartile range of these distribution metrics across all voxels are reported in Supplementary Figure S2-A. Consistent with a well-calibrated normative model, across all voxels, Z-scores in the 30% HC test set were approximately symmetric, centered close to zero, and had variances close to 1.0. Skewness and kurtosis were also close to zero. In addition, voxel-wise Shapiro–Wilk and Kolmogorov–Smirnov tests were performed across the whole brain on the Z-scores in the 30% HC test set to assess the normality of Z-scores. The resulting test statistics (W, D, and corresponding *p* values) were summarized across all voxels using the median and interquartile range (Supplementary Figure S2-B). For most voxels, the normality assumption was not rejected. The overall distribution of Z-scores was further examined using Q-Q plot and histogram based on the pooled Z-scores across all voxels and the 30% HC test participants (Supplementary Figure S3), which showed an overall distribution close to normal with only minor tail deviations. Formal normality tests were not applied to the pooled Z-scores because the extremely large sample size (voxel × participant) would make such tests overly sensitive to trivial deviations that are not practically meaningful [38, 39].

Together, the CV results and the normality checks of the held-out Z-scores support that the voxel-wise normative models were well fitted and suitable for deviation inference. The CV and train (70% HC)-test (30% HC) evaluation were conducted independently using the same predefined model settings, including the ‘WarpSinArcsinh’ warping function, reparameterization enabled and the L-BFGS-B optimizer, following a previous warped BLR study [40]. The CV procedure was used solely for model evaluation, and no hyperparameter tuning or model selection was performed. Therefore, no information from the CV (e.g., model fit parameters or hyperparameters) was carried over to the train (70% HC)-test (30% HC) evaluation. After validation, we retained the predefined model settings and refitted the warped BLR model on the whole HC participants (*N* = 232). This final model was then used to generate voxel-wise predictions and NPMs for all the IGD participants. This train–validate–refit procedure follows common practice in normative modeling studies, e.g., [16].

Lastly, to identify brain voxels showing significant deviations from healthy normative pattern in each participant, each individual NPM was thresholded using FDR (false discovery rate) correction at *p* < 0.01 [41]. Specifically, since the NPM (i.e., the Z-score map) is already a map with statistical measures (analogous to a t map), it can be subjected directly to multiple-comparison correction [16, 41]. Using the built-in function *y_FDR_Image.m* from DPABI v8.2, the Z-scores in each NPM were first converted to *p*-values, and FDR correction was then applied across voxels. After FDR thresholding, each participant’s NPM was binarized into positive and negative deviation masks (Z > 0 and Z < 0, respectively). To characterize the spatial distribution of deviations at the group level, we then generated overlap maps by counting, at each voxel, the number of participants exhibiting significant positive or negative deviations within the IGD and HC groups. Thus, the overlap maps reflect the prevalence of individual-level deviations across participants, rather than the magnitude of Z-scores, and were used to identify brain regions where deviations were most consistently observed in IGD relative to HC.

### Estimating regional deviations for each participant

To better characterize functionally meaningful deviations in each participant’s FC pattern, we derived regional deviation indices at the level of large-scale functional networks from the NPMs generated by the normative model. First, referring to [19, 36, 42], the proportion of voxels showing positive deviations (Z > 2) and the proportion of voxels showing negative deviations (Z < − 2) within each functional network were computed as regional deviations for every participant. These two quantities were treated as separate regional deviation indices, capturing the spatial extent of positive deviations and negative deviations in FC, respectively. Second, regarding large-scale functional networks, three network atlases were applied, i.e., the 10-network parcellation from Shen’s functional atlas (Shen10) [43, 44], the 17-network cortical parcellation by Yeo et al. (Yeo17) [45], and the 12-network parcellation derived from the Glasser 360-ROI atlas combined with the Ji 12-network partition (Glasser-Ji12) [46, 47].

To facilitate cross-template interpretation, we used the Network Correspondence Toolbox (NCT) [48] (implemented in Python3.11) to quantify overlap between Shen10 and the networks defined in Yeo17 and Glasser-Ji12. NCT computes overlap-based similarity (Dice coefficient) between binary network masks and evaluates significance using a spin-test–based permutation procedure. As shown in supplementary Figure S4, this analysis demonstrated robust and interpretable correspondence among conceptually similar large-scale networks across templates. In sum, for each participant’s NPM under each atlas, network-level positive- and negative-deviation indices for every functional network were computed, which were then used as input features for the clustering analyses.

### Exploratory clustering analyses

To exploratorily characterize heterogeneity in FC deviations within the IGD group, we next applied data-driven clustering to the regional deviation indices. To evaluate the robustness of any partitioning patterns across data, network templates, and clustering algorithms, we performed clustering separately for each data–atlas–algorithm combination. First, we conducted clustering analyses in three data: all the IGD participants, the out-of-sample 30% HC test subset, and the combined IGD + out-of-sample 30% HC participants data. For each data, each participant was represented by a feature vector comprising the concatenated deviation indices across networks, namely the proportion of positive suprathreshold voxels (Z > 2) and the proportion of negative suprathreshold voxels (Z < − 2) within each network. Prior to clustering, all features were z-scored across participants within each data to ensure comparable scaling across networks. To ensure comparability across data, the NPMs used for clustering in both all the IGD participants and the 30% HC participants were all derived from the same normative model trained on the 70% HC training subset.

Second, the above feature construction and clustering were performed separately under each of three large-scale network parcellations: Shen10, Yeo17, and Glasser-Ji12. Thus, for every data, we generated atlas-specific feature spaces reflecting network-level deviations defined by that atlas, enabling us to assess whether any clustering-based stratification pattern was consistent across commonly used atlas choices. Third, within each data–atlas feature space, we fit clustering models using two unsupervised algorithms: Gaussian mixture modeling (GMM) [49] implemented in Python3.11 using *mixture.GaussianMixture* from *sklearn* and K-means clustering [50] implemented in Python3.11 using *cluster.KMeans* from *sklearn*. For each algorithm, we varied the number of clusters (K) from 2 to 10 and, for every candidate K, obtained cluster assignments and computed the silhouette coefficient to quantify within-cluster cohesion and between-cluster separation [51], implemented in Python3.11 using *metrics.silhouette_score* from *sklearn*. The value of K that maximized the silhouette score was selected as the primary solution for that specific data–atlas–algorithm combination. In sum, clustering was performed in a fully factorial manner (Supplementary Table S1) across the three data, three network atlases, and two algorithms, with the optimal number of clusters selected independently for each data–atlas–algorithm combination.

## Results

### Individual deviation from normative models

**Fig. 2:**
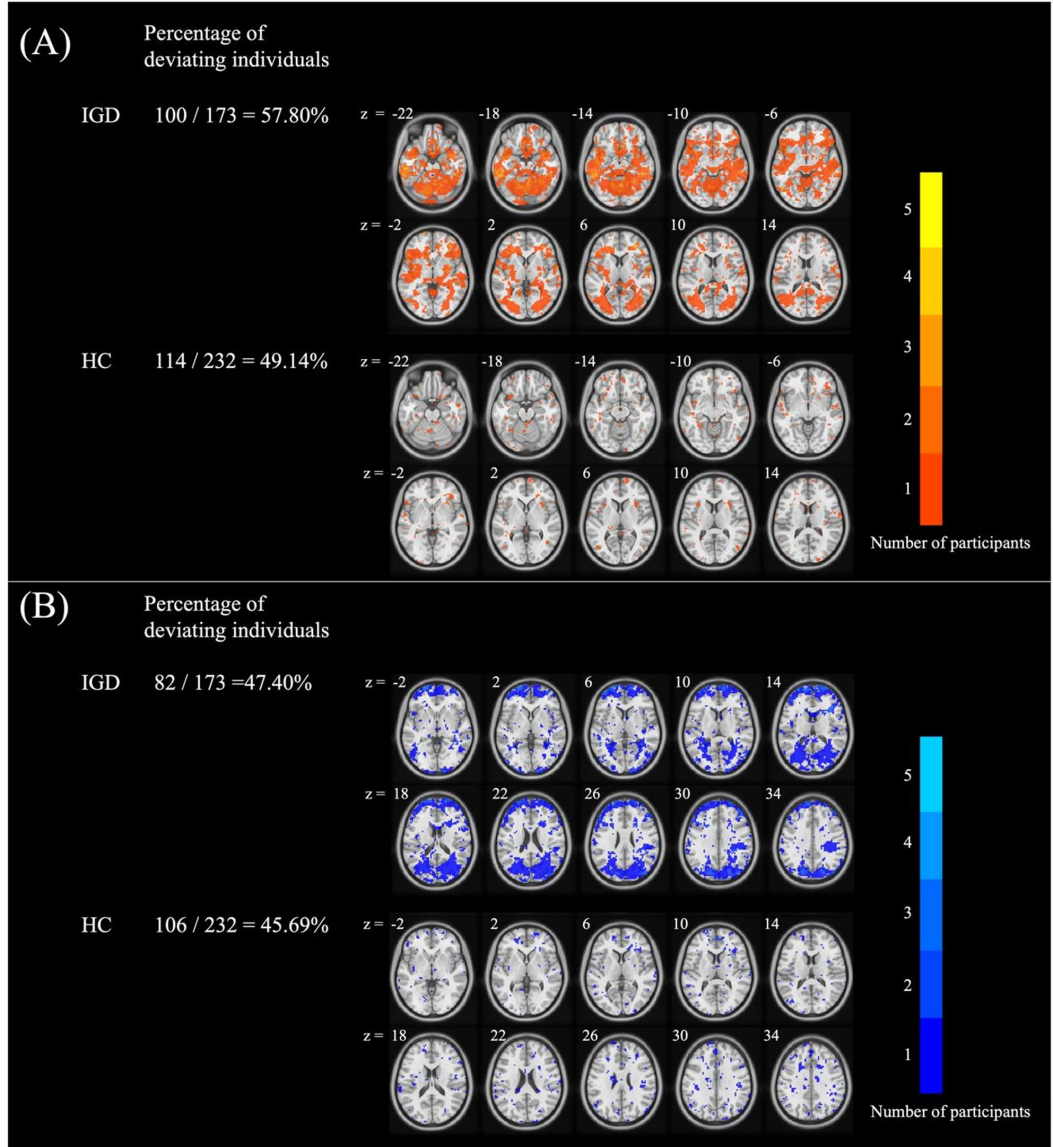
Overlap of significant positive and negative deviation masks from FDR-corrected NPMs in the IGD and HC groups (axial slices). (**A**). Overlap of significant positive deviation masks from FDR-corrected NPMs in the IGD and HC groups. (**B**). Overlap of significant negative deviation masks from FDR-corrected NPMs in the IGD and HC groups. Notes: The “Percentage of deviating individuals” denotes the proportion of participants in each group who show at least one significant deviant voxel (i.e., who have any suprathreshold voxels). Voxel values indicate the number of participants showing a significant deviation at each voxel. Abbreviations: IGD, Internet Gaming Disorder; HC, Healthy Control; FDR, False Discovery Rate; NPMs, Normative Probability Maps

Figure 2 shows group-level overlap maps generated by overlaying the binarized (positive/negative) deviation masks derived from each participant’s FDR-corrected NPM. Specifically, Fig. 2 indicates the number of participants exhibiting significant positive or negative deviations at each voxel relative to the normative model, shown separately for the IGD and HC groups. As stated in Methods, for the IGD participants, their NPMs were generated based on the voxel-wise normative model trained on the whole HC participants. For the HC participants, each individual appeared in the test set exactly once during the 10-fold CV, and their NPMs were generated in the fold in which they belonged to the held-out 10% test set, using the voxel-wise normative model trained on the remaining 90% of the HC participants. Thus, the NPMs for the IGD participants and the HC participants are essentially comparable, as they are all deviation scores obtained when each participant served as a test case. Under the null hypothesis that the IGD participants follow a similar trajectory of brain FC as the HC participants, there is no prior reason to expect that the fit will be better in the HC participants than in the IGD participants [16]. However, as shown in Fig. 2, the HC group showed few significant positive and negative deviations. In contrast, the voxels with significant deviations in the IGD group were noticeably more than in the HC group and were widespread across the brain.

**Fig. 3:**
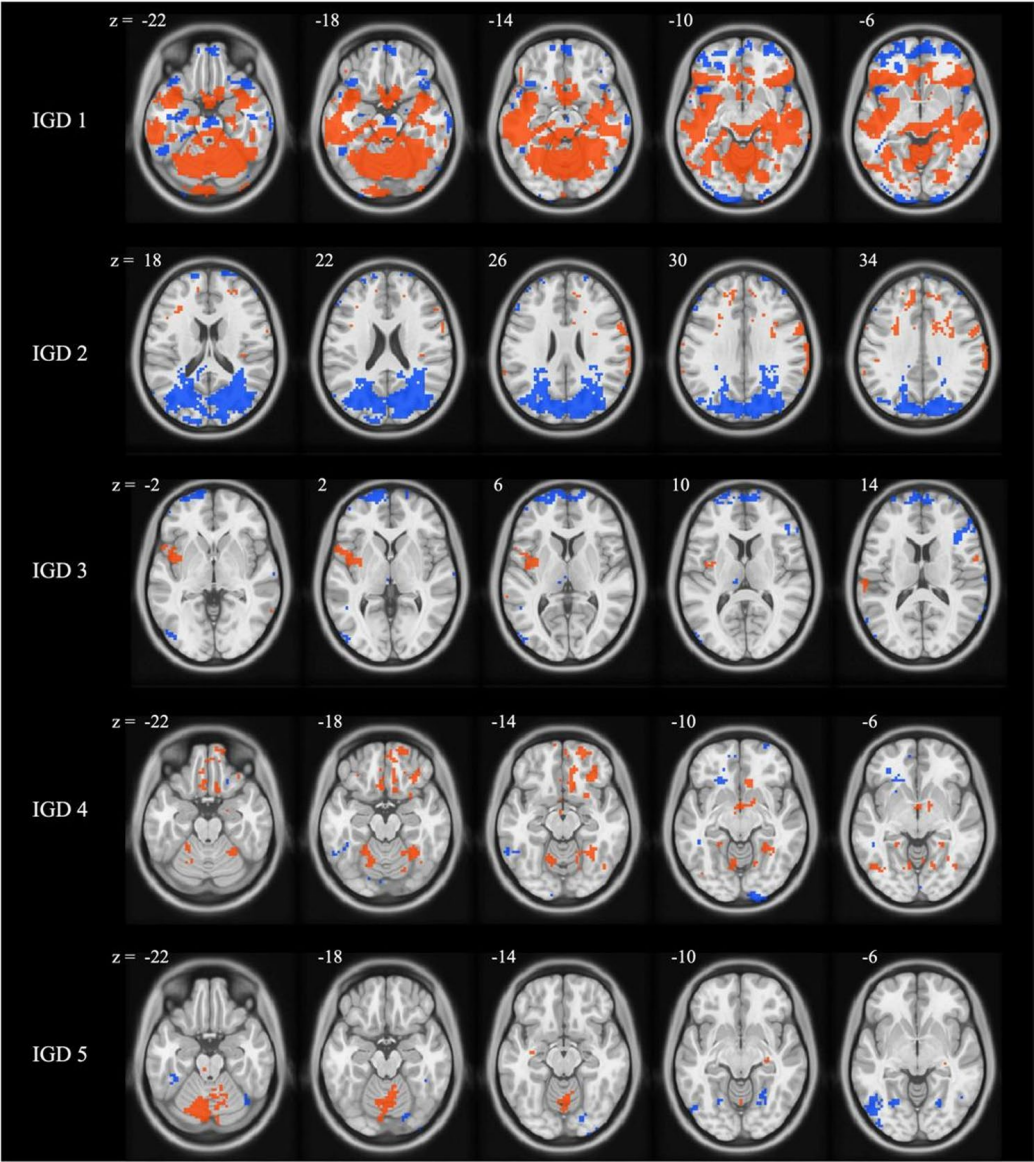
Individual NPM deviation masks after FDR correction: five IGD participants with the largest number of significant deviation voxels (axial slices). Notes: Red indicates voxels with significant positive deviations, whereas blue indicates voxels with significant negative deviations. Abbreviations: IGD, Internet Gaming Disorder; NPM, Normative Probability Map; FDR, False Discovery Rate

Figure 3 shows the binarized, FDR-corrected NPMs of five IGD participants with the largest number of significantly deviant voxels in their NPMs (i.e., ranked by the voxel count surviving FDR correction). These individuals represented only a small fraction of the IGD group, and their deviation patterns differed markedly from one another. Together, these results highlight substantial inter-individual variability within IGD and indicate that such variability should be considered when interpreting group-level findings.

### Comparison between normative modeling and case-control

**Fig. 4:**
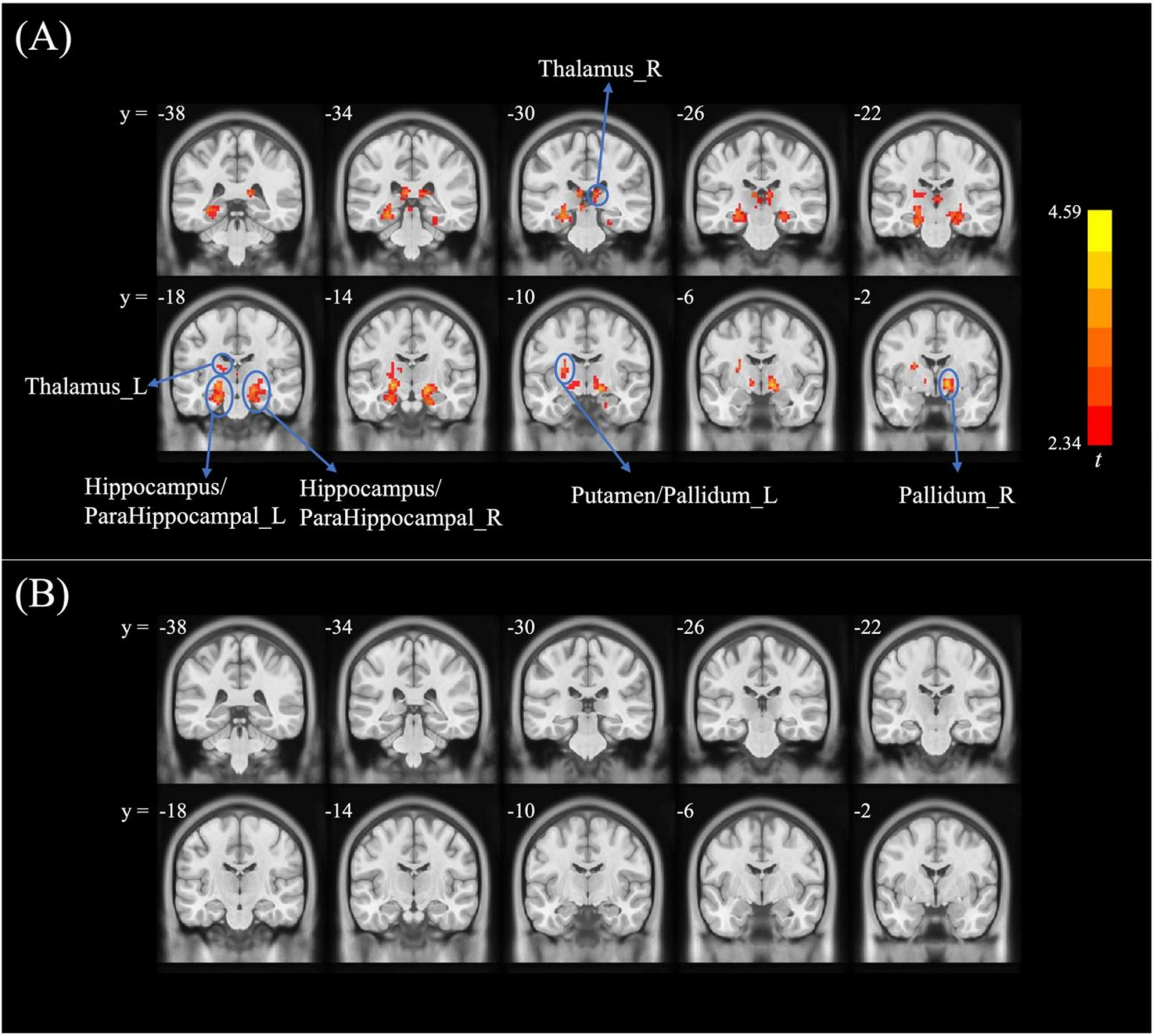
The results of case-control method (axial slices). (**A**). The significant difference of FC between the IGD group and the HC group after using FWE correction with voxel *p* = 0.01 and cluster *p* = 0.05. (**B**). No significant difference of FC between the IGD group with removing the top 10 IGD participants with the largest number of deviated voxels surviving FDR correction in NPMs and the HC group, after using FWE correction with voxel *p* = 0.01 and cluster *p* = 0.05. Abbreviations: IGD, Internet Gaming Disorder; HC, Healthy Control; L, Left; R, Right; FC, Functional Connectivity; FWE, Family-Wise Error; NPM, Normative Probability Map

Using a conventional case–control approach, we performed a voxel-wise independent-samples t-test on the seed-to-voxel FC maps (Fisher r-to-z transformed) to compare the IGD and HC groups, i.e., comparing between-group differences in mean FC at each voxel in the whole brain. Statistical significance was assessed using family-wise error (FWE) correction with a voxel-level threshold of *p* = 0.01 and a cluster-level threshold of *p* = 0.05 to identify regions showing significant between-group differences in NAcc-based FC. The results (Fig. 4-A) showed that the IGD group had significantly stronger FC between the bilateral NAcc and medial temporal regions (primarily involving the hippocampus and parahippocampal gyrus), with extensions into subcortical regions including the putamen, pallidum, and thalamus, than the HC group. Anatomical labels were assigned based on the Automated Anatomical Labeling 3 (AAL3) [52].

Then, we removed the top 10 IGD participants with the largest number of deviant voxels surviving FDR correction in their NPMs (i.e., ranked by the FDR-corrected voxel count) and re-ran the independent-samples t-test on seed-to-voxel FC maps (Fisher r-to-z transformed) between the remaining IGD participants (*N* = 163) and the HC group. The same FWE correction (voxel-level *p* = 0.01 and cluster-level *p* = 0.05) was also applied. As shown in Fig. 4-B, no significant between-group difference was detected.

### The results of exploratory clustering analyses

Across all clustering analyses, spanning all data, atlases, and algorithms, the silhouette criterion consistently selected an optimal solution of K = 2 clusters (Figure S5, S6 and S7). Because the silhouette coefficient is not defined for K = 1, the primary clustering analyses for both GMM and K-means evaluated candidate solutions from K = 2 to K = 10. As GMM allows formal evaluation of a single-cluster solution, we further compared GMM models with K = 1 versus K = 2 using the Bayesian information criterion (BIC) as an auxiliary check on whether a single-cluster solution might provide a more appropriate description of the data. Lower BIC values indicate a more favorable clustering solution after accounting for model complexity. Across all data–atlas combinations, BIC values were lower for K = 2 than for K = 1 (Supplementary Table S2).

**Fig. 5:**
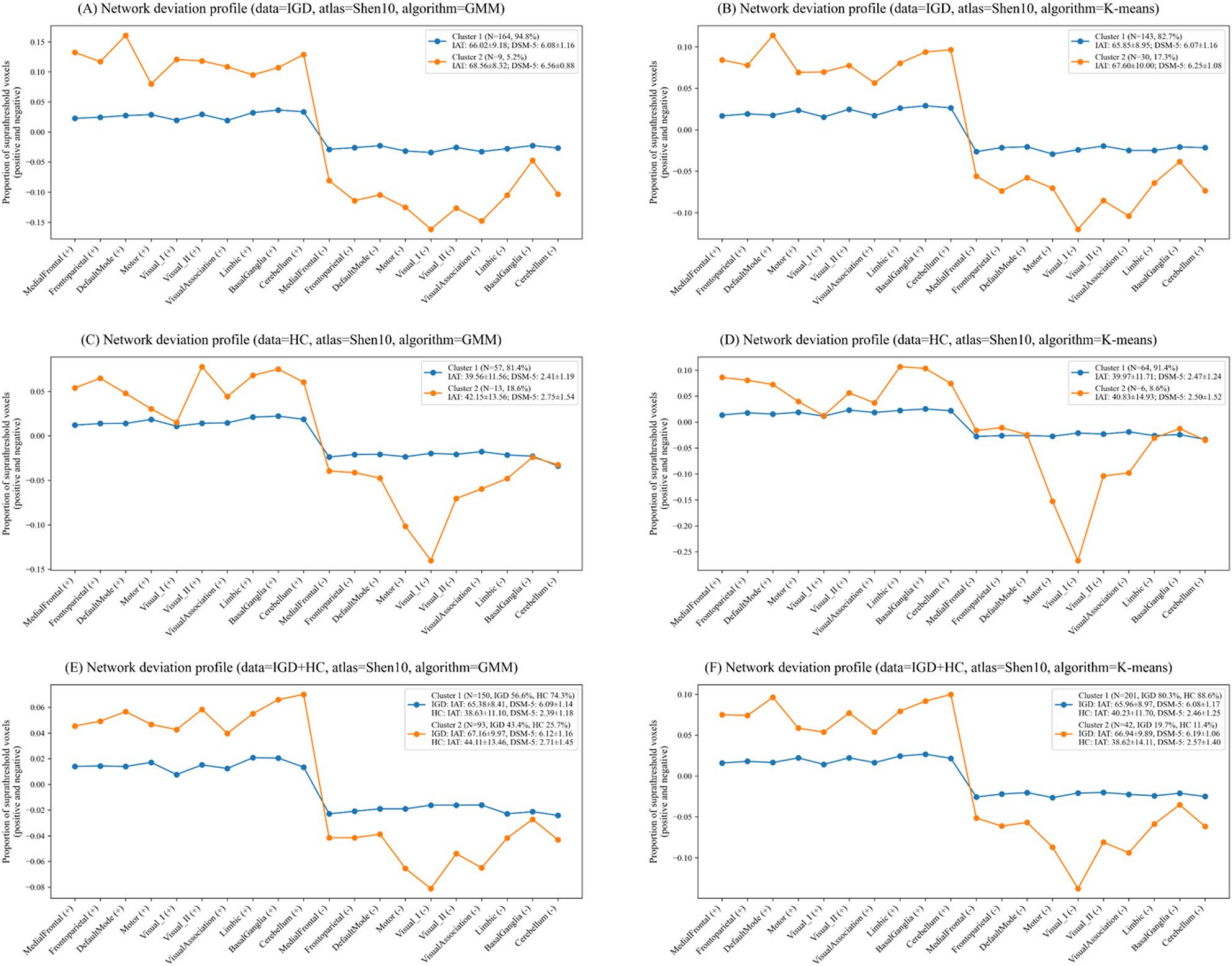
Cluster-wise network deviation profiles under the Shen10 atlas (three data × two algorithms). Notes: Three data: “IGD” —all the IGD participants data, “HC” —the out-of-sample 30% HC participants data, and “IGD + HC” —the combined IGD + out-of-sample 30% HC participants data; Two algorithms: GMM and K-means. The “(+)” and “(−)” symbols shown in the x-axis labels indicate positive deviations (Z > 2) and negative deviations (Z < − 2), respectively. Accordingly, the proportion of suprathreshold positive deviations within each network is plotted as a positive value, whereas the proportion of suprathreshold negative deviations is plotted as the negative of that proportion (i.e., multiplied by − 1). This sign convention is used purely for visualization, to clearly distinguish positive from negative deviations within a single profile. The legend reports, for each cluster, the cluster size and percentage, as well as the mean ± SD of IAT and DSM-5 scores (computed within the corresponding group when applicable). In particular, for the combined IGD + HC data, the IGD% and HC% shown are within-group percentages (i.e., relative to the total number of IGD or HC participants, respectively), rather than percentages of the total cluster size, to more clearly show how many individuals from each group (IGD vs. HC) were assigned to each cluster. Abbreviations: IGD, Internet Gaming Disorder; HC, Healthy Control; IAT, Internet Addiction Test; DSM-5, the fifth edition of Diagnostic and Statistical Manual of Mental Disorders; GMM, Gaussian Mixture Modeling; Shen10, 10-network parcellation from Shen’s functional atlas

In the IGD-only, HC-only, and combined IGD + HC data, these two clusters exhibited a highly similar qualitative pattern across methods and templates (Fig. 5, Figures S8 and S9): one cluster included the majority of participants and was characterized by generally low proportions of suprathreshold voxels across networks (a “low-deviation” stratum), whereas the second cluster comprised a smaller subset of individuals showing higher proportions of deviations across multiple networks (a “high-deviation” stratum). The same low- vs. high-deviation stratification pattern emerged consistently, with variation mainly in stratum prevalence rather than in the overall pattern. In addition, all GMM solutions converged successfully, and the K-means solutions converged well before the prespecified iteration limit. Repeated runs under different random initializations yielded generally high adjusted Rand index (ARI) values (most > 0.90), which quantify the consistency of participant assignment across repeated clustering runs, indicating that the resulting partitions were relatively stable with respect to initialization. Together, these results suggest that the identified two-stratum partition was reproducible across analytic choices at an exploratory level. However, this pattern does not provide evidence for discrete subtypes and may instead be compatible with a continuous distribution of deviations across individuals. Importantly, although the HC group could also be partitioned into two clusters with the same low- vs. high-deviation stratification pattern, the fraction of HC participants assigned to the high-deviation stratum was clearly smaller than the corresponding fraction of IGD participants in the high-deviation stratum. The corresponding numerical values are provided in Supplementary Tables S3, S4 and S5.

To further examine the possible clinical relevance of the clustering pattern, we performed Spearman rank correlation analyses within the IGD group, including correlations between IAT/DSM-5 scores and a global deviation measure (defined as the mean value across all network-level deviation features), as well as feature-wise correlations between IAT/DSM-5 scores and each individual network-level deviation measure (FDR corrected). These analyses yielded uniformly negative results, with no significant associations detected within the IGD group. Although in most clustering solutions the higher-deviation stratum tended to show numerically higher IAT and DSM-5 scores than the lower-deviation stratum, these differences were small and were not supported by the continuous correlation analyses.

## Discussion

### The heterogeneity in abnormal FC of IGD participants

Our results provide compelling evidence that IGD is characterized by substantial heterogeneity in resting-state FC. The normative modeling approach revealed that a minority of IGD individuals exhibited widespread and diverse deviations in FC between the bilateral NAcc and the remaining brain, whereas the majority exhibited fewer and still diverse deviations. The diversity was evident from the overlap of all the IGD participants’ FDR-corrected deviation maps (Fig. 2). Only a small fraction of voxels showed deviations shared by as many as five participants, whereas for most voxels, deviations were observed in only one or two participants. In addition, as shown in Fig. 3, the spatial distribution and direction (positive vs. negative) of deviant voxels varied across participants. This is consistent with prior findings in psychiatric disorders such as ASD, major depressive disorder, and bipolar disorder, where patients show heterogeneous deviations across the brain in neural features, e.g., gray matter volume and cortical thickness, and only a small portion of patients show large deviations [14, 16, 20, 21]. Such inter-individual variability in FC deviations in IGD, together with methodological differences across studies (e.g., sample composition, preprocessing choices, and statistical thresholds), may contribute to the variability of findings in the prior IGD RS-fMRI literature, as reviewed in the Background. Moreover, when inter-individual variability is marked, case–control analyses that average across individuals may be disproportionately influenced by a subset of participants, yielding group differences that are not uniformly expressed across the entire group.

Indeed, our results suggests that conventional group-mean contrasts can be shaped by a small subset of individuals with more extreme deviations. In the present study, our conventional case–control analysis yielded significant between-group FC differences (e.g., increased NAcc–medial temporal regions and parts of subcortical regions connectivity in IGD than in HC), but these effects became non-significant after removing a very small number (≈ 6%) of particularly deviant IGD individuals. This pattern suggests that at least some apparent “group differences” may be disproportionately influenced by a minority of extreme cases, which can inflate or distort conclusions about disorder-level abnormalities when individual variability is ignored. At the same time, the loss of statistical significance after exclusion should be interpreted cautiously, because it could also reflect reduced power due to the smaller sample, the stringency of voxel-wise multiple-comparison correction, or genuinely small group-mean effects in this FC contrast. Additionally, similar observations have been reported in ASD. After removing a small subset of extremely deviant participants, the number of cortical regions showing between-group differences in cortical thickness between the ASD and HC groups was reduced by half [19]. Together, these findings highlight the importance of complementing traditional group comparisons with individualized deviation mapping when the underlying neurobiology is heterogeneous and suggest that normative modeling can help characterize participant-specific FC deviations that may be obscured—or appear unstable—under averaged group contrasts, consistent with broader precision-medicine perspectives in psychiatry [53, 54].

### Exploratory deviation stratification and potential implications

To move from voxel-level idiosyncrasy toward a more interpretable description of inter-individual heterogeneity, we summarized deviations at the level of large-scale functional networks and applied unsupervised clustering across data, atlases, and algorithms. Across these exploratory clustering analyses, the silhouette criterion consistently selected an optimal solution of K = 2 clusters. This convergence across analytic choices suggests that the dominant structure of individual differences in our deviation features is reliably captured by two strata, and that the resulting stratification is robust to commonly used parcellations and clustering method [55]. Conceptually, the two clusters reflected a “low-deviation” stratum comprising most participants and a smaller “high-deviation” stratum showing broadly elevated proportions of suprathreshold deviations across networks, a pattern that echoes normative-model findings in other psychiatric conditions where heterogeneity is often expressed as a minority of individuals with widespread deviations [14, 19]. The clustering results also help interpret the overlap maps: although few voxels showed deviations shared by many individuals, indicating substantial voxel-level heterogeneity, these idiosyncratic patterns could still be summarized into a more stable network-level description of heterogeneity [14, 21, 56].

At the same time, the present results do not support interpreting these strata as discrete neurobiological subtypes. A similar low- vs. high-deviation partition could also be observed in the HC data, although the proportion of participants assigned to the higher-deviation stratum was smaller than in the IGD data, suggesting that such a pattern is not unique to IGD but is more prevalent in IGD. Moreover, the correlation analyses within the IGD group did not reveal significant relationships between deviation measures and IAT/DSM-5 scores. Thus, the current findings are better understood as an exploratory higher- vs. lower-deviation stratification, rather than evidence for distinct subtype structure. Nevertheless, in most clustering solutions, the higher-deviation stratum showed numerically slightly higher IAT and DSM-5 scores than the lower-deviation stratum, which may provide a tentative clue for future work. From a clinical perspective, this pattern may still offer an exploratory way to organize heterogeneity in IGD, although its clinical utility remains to be established. Future studies with larger samples, longitudinal follow-up, and multimodal measures will be needed to determine whether this deviation-based stratification is associated with symptom trajectories, relapse risk, treatment response, or other clinically meaningful outcomes.

### Limitations

Four limitations should be noted. First, although we assessed robustness across data, atlases, and algorithms, all clustering analyses were conducted within a single study cohort. Independent replication in external samples (with broader age ranges, clinical severity, and comorbidity profiles) will be essential to confirm the prevalence and interpretability of the low- vs. high-deviation strata in IGD. Second, we focused on NAcc seed-to-voxel resting-state FC. IGD likely involves distributed alterations beyond NAcc-based connectivity, and future work should extend normative modeling to whole-connectome features, additional seeds, and/or dynamic FC to better capture multidimensional heterogeneity. Third, because the data are cross-sectional, we cannot determine whether the observed deviations are predisposing factors, consequences of prolonged gaming, or state-dependent correlates. Fourth, a small number of participants in the HC group exceeded one screening threshold (IAT or DSM-5) but not both. Given that they did not meet the full criteria for IGD, they were retained as comparison participants. Future studies with more stringently defined HC samples will be helpful for validating the present findings.

## Conclusions

The present study provides two main findings about resting-state FC in IGD. First, using normative modeling, we observed marked inter-individual heterogeneity in NAcc-centered FC deviations: a minority of IGD participants showed more widespread deviations, whereas most participants exhibited fewer deviating voxels with highly variable spatial patterns. Group-level overlap maps further indicated that deviations were rarely shared across many individuals, underscoring the idiosyncratic nature of voxel-wise deviation patterns. Second, when deviations were summarized at the level of large-scale functional networks, unsupervised clustering yielded a robust and parsimonious higher- vs. lower-deviation stratification across sample data, atlas templates, and clustering algorithms. Together, these findings highlight the value of individualized deviation mapping and network-level summaries for characterizing heterogeneity in IGD, while suggesting that the observed stratification should be interpreted as exploratory rather than as evidence for discrete subtypes.

## Electronic Supplementary Material

Below is the link to the electronic supplementary material.

Supplementary Material 1

Supplementary Material 2

## Data Availability

The data generated and analyzed during the current study are not publicly available due to restrictions related to participant privacy and institutional ethical regulations, but are available from the corresponding author on reasonable request.

## Abbreviations

IGD: Internet gaming disorder
APA: American psychiatric association
DSM-5: Fifth edition of diagnostic and statistical manual of mental disorders
RS-fMRI: Resting-state functional magnetic resonance imaging
HC: Healthy control
FC: Functional connectivity
DLPFC: Dorsolateral prefrontal cortex
NAcc: Nucleus accumbens
OFC: Orbitofrontal cortex
ASD: Autism spectrum disorder
BLR: Bayesian linear regression
ROI: Region of interest
IAT: Internet addiction test
DPABI: Data processing & analysis for brain imaging
EPI: Echo-planar imaging
TR: Repetition time
TE: Echo time
FOV: Field of view
MNI152: Montreal neurological institute152
FWHM: Full width at half maximum
PCNtoolkit: Predictive clinical neuroscience toolkit
CV: Cross-validation
EV: Explained variance
MSLL: Mean standardized log loss
NPM: Normative probability map
FDR: False discovery rate
Shen10: 10-network parcellation from Shen’s functional atlas
Yeo17: 17-network cortical parcellation by Yeo et al
Glasser-Ji12: 12-network parcellation derived from the Glasser 360-ROI atlas combined with the Ji 12-network partition
NCT: Network correspondence toolbox
GMM: Gaussian mixture modeling
FEW: Family-wise error
AAL3: Automated anatomical labeling3
BIC: Bayesian information criterion
ARI: Adjusted rank index
