# Supplementary material for: Normative modeling reveals functional connectivity heterogeneity in internet gaming disorder
Source: Ann Gen Psychiatry. 2026 May 2;25:50. doi: 10.1186/s12991-026-00664-3 (PMC13285037; doi:10.1186/s12991-026-00664-3)
Supplement: Supplementary file 1 — Supplementary Material 1 [file 12991_2026_664_MOESM1_ESM.docx]

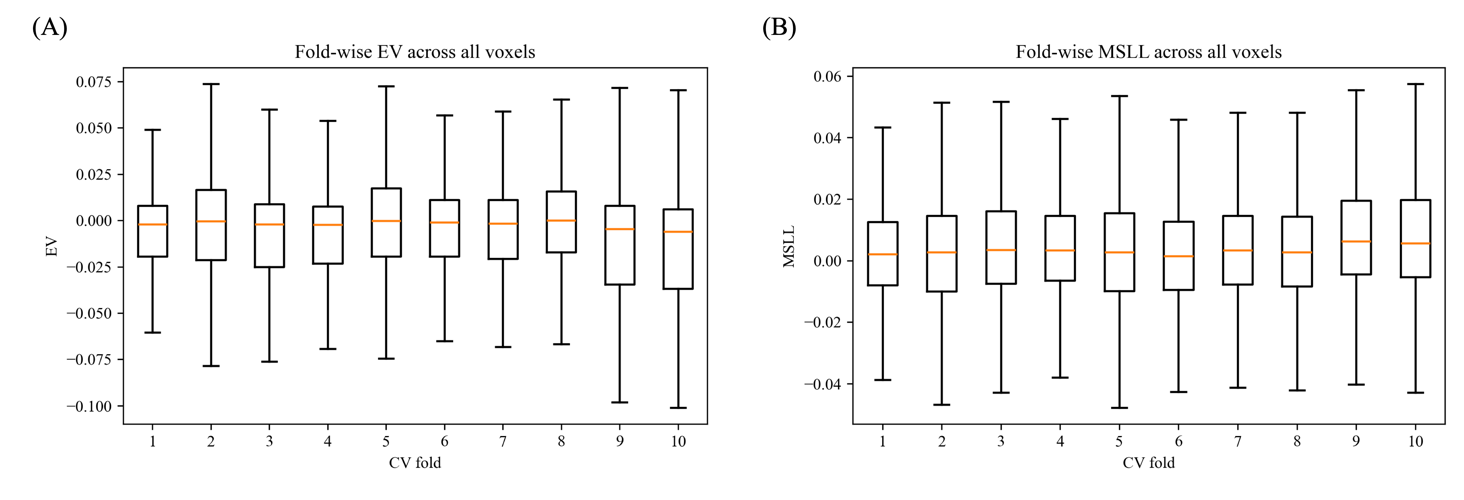


**Figure S1 Fold-wise EV and MSLL across all voxels**

(A) Median and interquartile range of EV across all voxels for each of the 10 folds.

(B) Median and interquartile range of MSLL across all voxels for each of the 10 folds.

Abbreviations: CV, cross-validation; EV, explained variance; MSLL, mean standardized log loss.


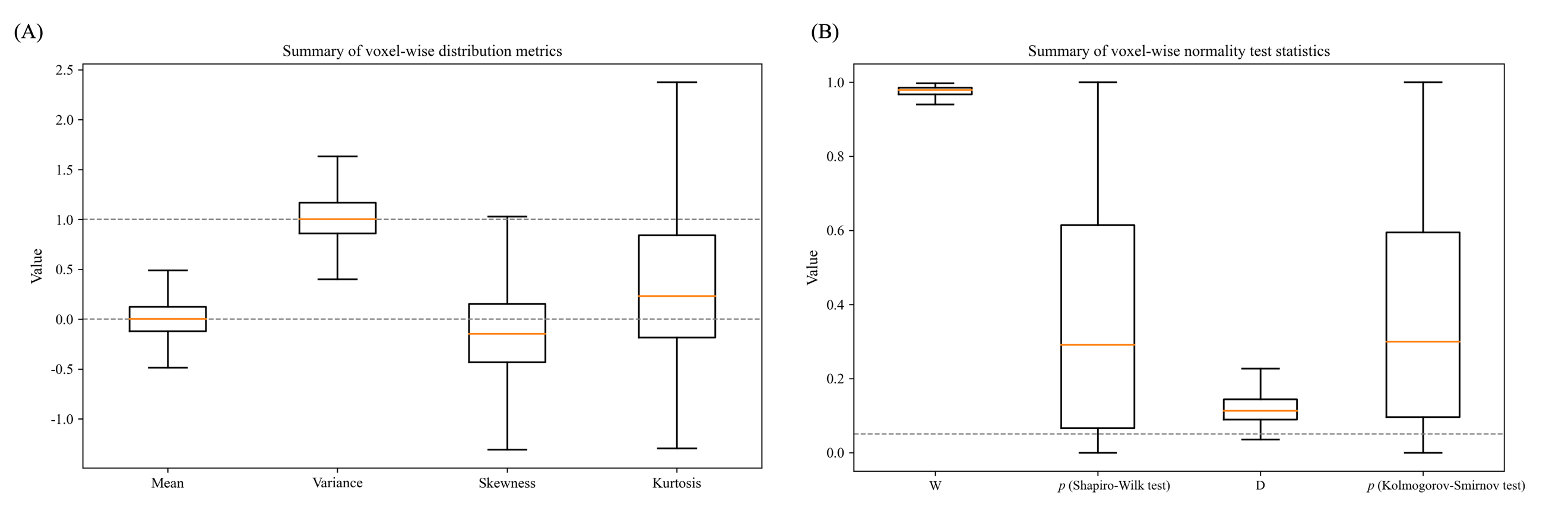


**Figure S2** **Median and interquartile range of** **distribution metrics and normality test statistics across all voxels**

(A) Median and interquartile range of distribution metrics across all voxels. Distribution metrics include the voxel-wise mean, variance, skewness, and kurtosis of Z-scores in the 30% HC test set.

(B) Median and interquartile range of normality test statistics across all voxels. Normality test statistics include the voxel-wise W and corresponding *p* values from the Shapiro-Wilk test, as well as the D and corresponding *p* values from the Kolmogorov-Smirnov test, computed based on the Z-scores in the 30% HC test set.

Abbreviations: HC, Healthy Control.

**
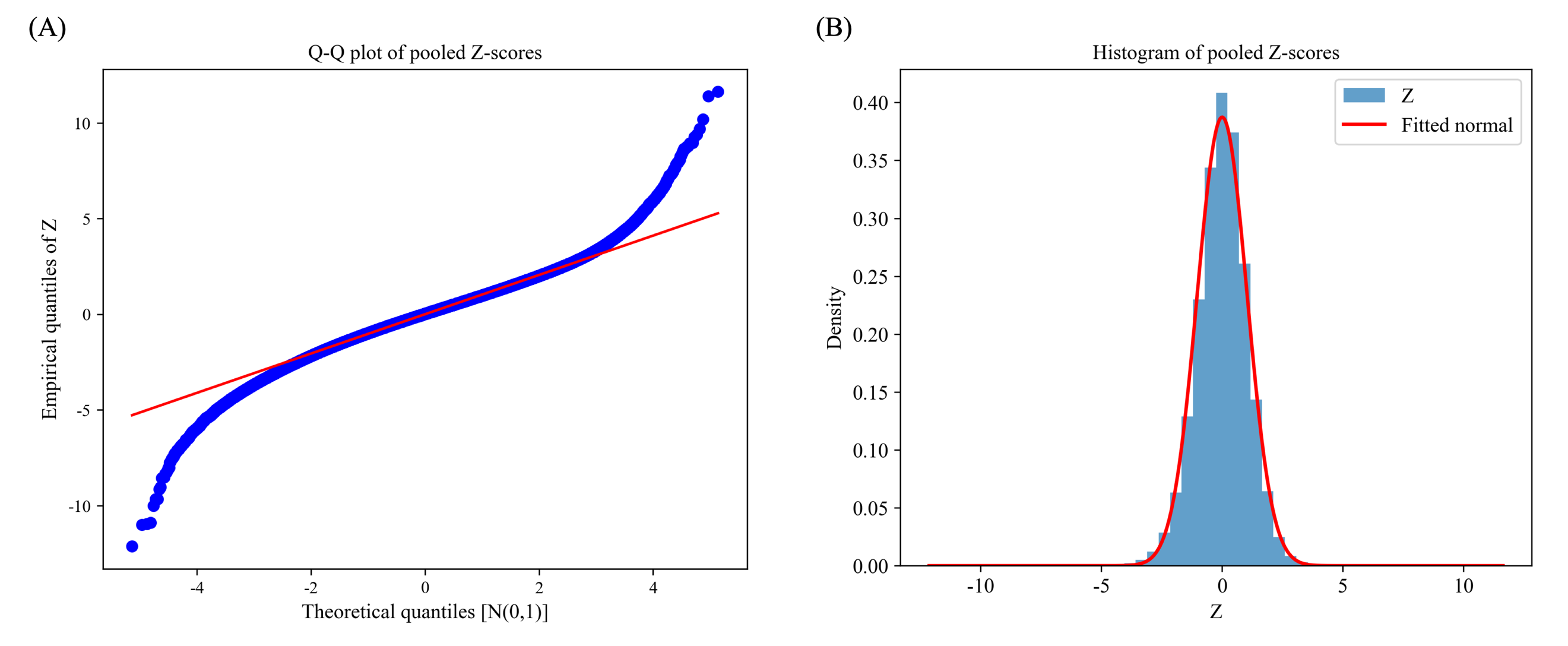
**

**Figure S3 Q-Q plot and histogram of pooled Z-scores**

(A) Q-Q plot of pooled Z-scores across all voxels and the 30% HC test participants.

(B) Histogram of pooled Z-scores across all voxels and the 30% HC test participants.

Abbreviations: HC, Healthy Control.


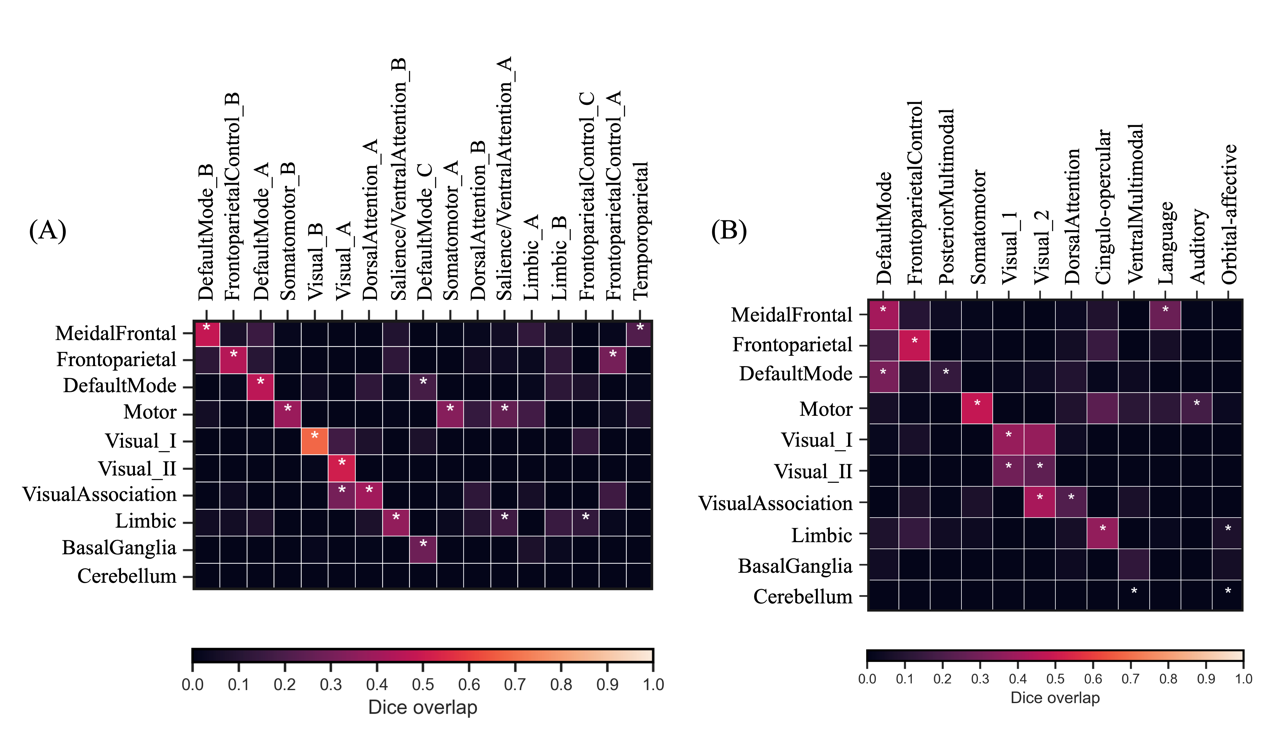


**Figure S4 Quantified overlap between Shen10 and the networks defined in Yeo17 and Glasser-Ji12**

(A) The network correspondence between Shen10 and Yeo17, quantified as Dice-coefficient overlap between their binary network masks (with spin-test permutation *p*-values indicating whether overlap exceeds chance).

(B) The network correspondence between Shen10 and Glasser-Ji12, quantified as Dice-coefficient overlap between their binary network masks (with spin-test permutation *p*-values indicating whether overlap exceeds chance).

Abbreviations: * indicates *p*<0.05, a significant overlap between the two network regions. Shen10, 10-network parcellation from Shen’s functional atlas; Yeo17, 17-network cortical parcellation by Yeo et al; Glasser-Ji12, 12-network parcellation derived from the Glasser 360-ROI atlas combined with the Ji 12-network partition.

**
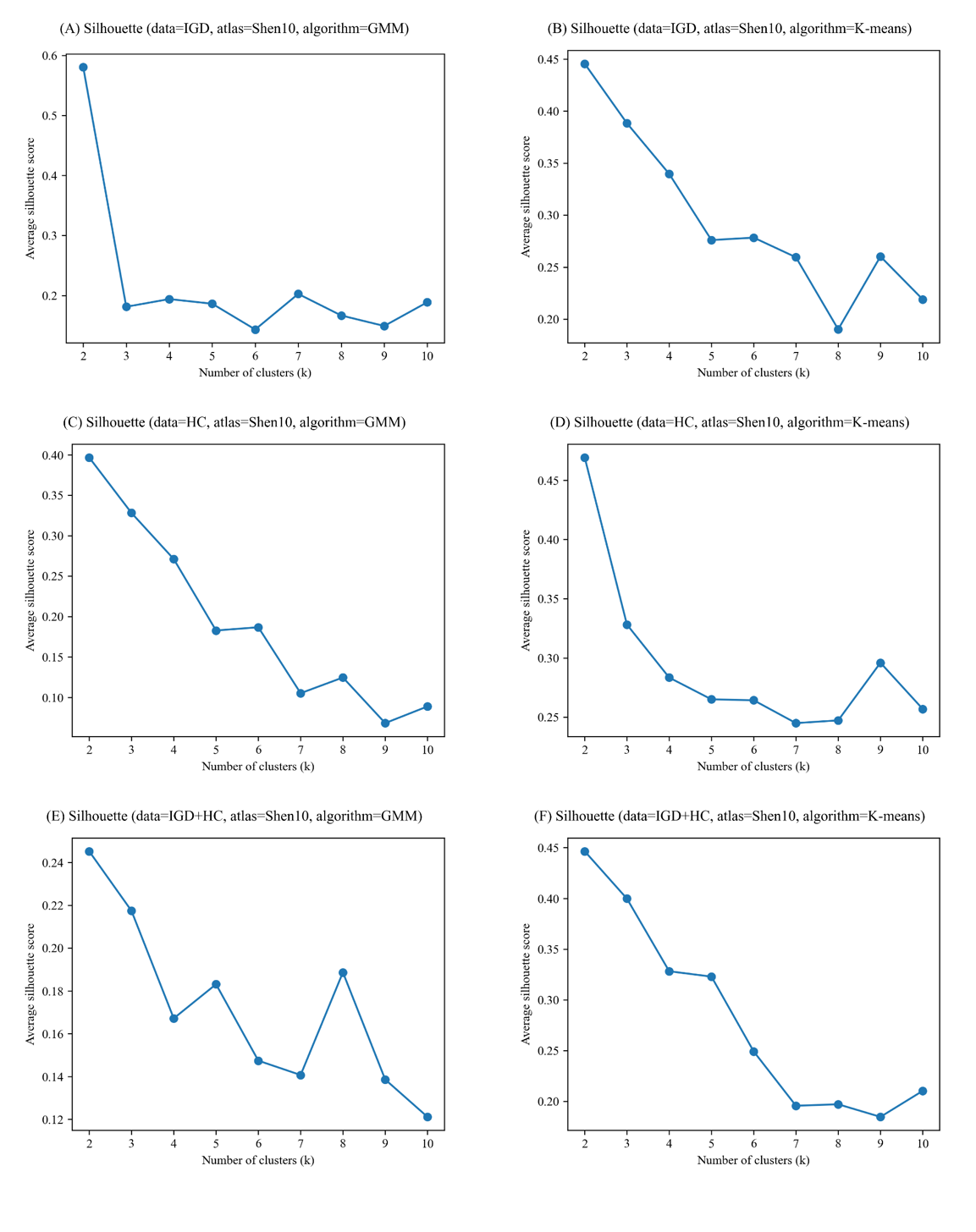
**

**Figure S5** **Silhouette analyses for selecting the optimal number of clusters under the Shen10 atlas (three data ×** **two algorithms)**

Notes: Three data: “IGD” —all the IGD participants data, “HC” —the out-of-sample 30% HC participants data, and “IGD+HC” —the combined IGD+out-of-sample 30% HC participants data; Two algorithms: GMM and K-means.

Abbreviations: IGD, Internet Gaming Disorder; HC, Healthy Control; GMM, Gaussian Mixture Modeling; Shen10, 10-network parcellation from Shen’s functional atlas.


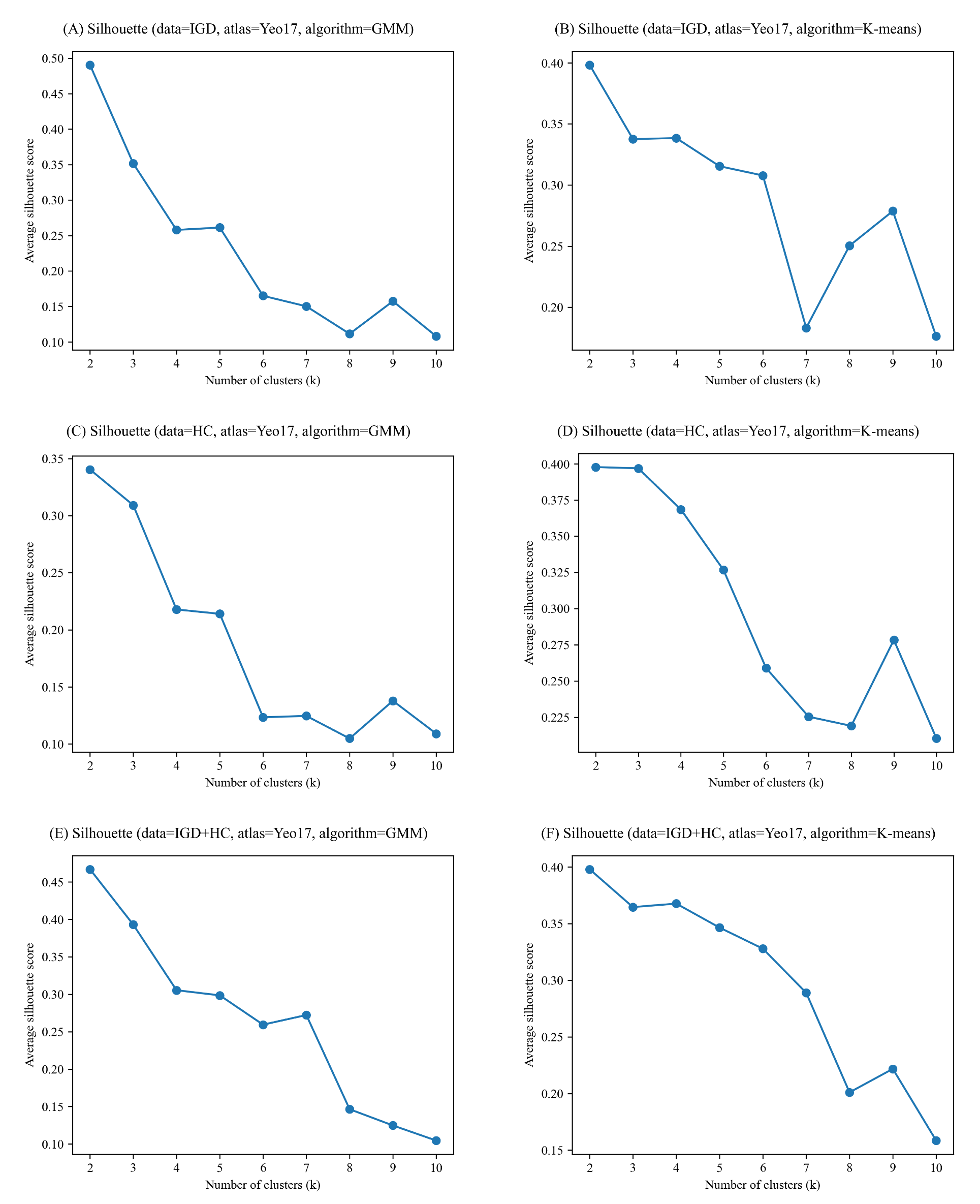


**Figure S6 Silhouette analyses for selecting the optimal number of clusters under the Yeo17 atlas (three data × two algorithms)**

Notes: Three data: “IGD” —all the IGD participants data, “HC” —the out-of-sample 30% HC participants data, and “IGD+HC” —the combined IGD+out-of-sample 30% HC participants data; Two algorithms: GMM and K-means.

Abbreviations: IGD, Internet Gaming Disorder; HC, Healthy Control; GMM, Gaussian Mixture Modeling; Yeo17, 17-network cortical parcellation by Yeo et al.


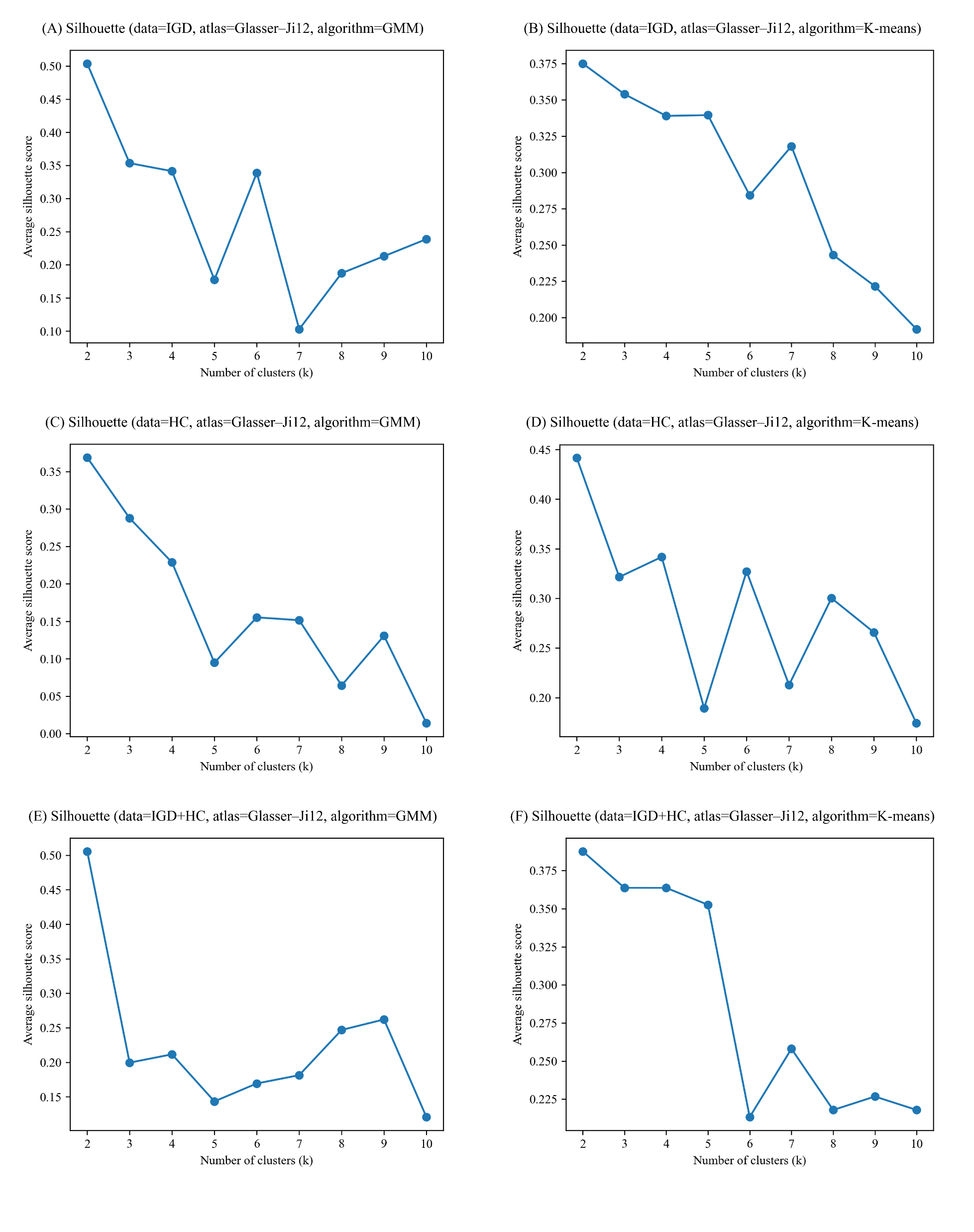


**Figure S7 Silhouette analyses for selecting the optimal number of clusters under the Glasser-Ji12 atlas (three data × two algorithms)**

Notes: Three data: “IGD” —all the IGD participants data, “HC” —the out-of-sample 30% HC participants data, and “IGD+HC” —the combined IGD+out-of-sample 30% HC participants data; Two algorithms: GMM and K-means.

Abbreviations: IGD, Internet Gaming Disorder; HC, Healthy Control; GMM, Gaussian Mixture Modeling; Glasser-Ji12, 12-network parcellation derived from the Glasser 360-ROI atlas combined with the Ji 12-network partition.


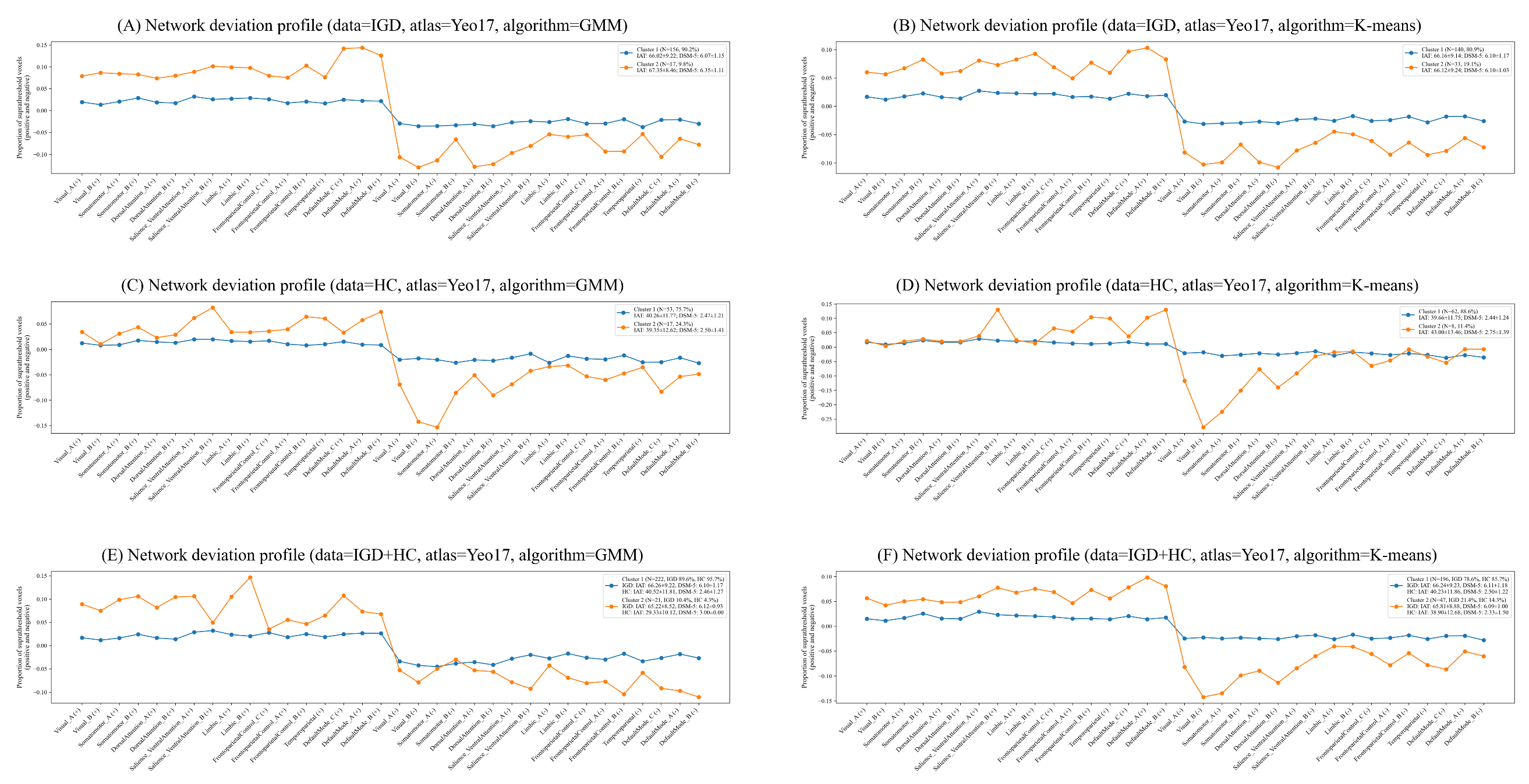


**Figure S8** **Cluster-wise network deviation profiles under the Yeo17 atlas (three data × two algorithms)**

Notes: Three data: “IGD” —all the IGD participants data, “HC” —the out-of-sample 30% HC participants data, and “IGD+HC” —the combined IGD+out-of-sample 30% HC participants data; Two algorithms: GMM and K-means. The “(+)” and “(−)” symbols shown in the x-axis labels indicate positive deviations (Z > 2) and negative deviations (Z < −2), respectively. Accordingly, the proportion of suprathreshold positive deviations within each network is plotted as a positive value, whereas the proportion of suprathreshold negative deviations is plotted as the negative of that proportion (i.e., multiplied by −1). This sign convention is used purely for visualization, to clearly distinguish positive from negative deviations within a single profile. The legend reports, for each cluster, the cluster size and percentage, as well as the mean ± SD of IAT and DSM-5 scores (computed within the corresponding group when applicable). In particular, for the combined IGD+HC data, the IGD% and HC% shown are within-group percentages (i.e., relative to the total number of IGD or HC participants, respectively), rather than percentages of the total cluster size, to more clearly show how many individuals from each group (IGD vs. HC) were assigned to each cluster.

Abbreviations: IGD, Internet Gaming Disorder; HC, Healthy Control; IAT, Internet Addiction Test; DSM-5, the fifth edition of Diagnostic and Statistical Manual of Mental Disorders; GMM, Gaussian Mixture Modeling; Yeo17, 17-network cortical parcellation by Yeo et al.

**
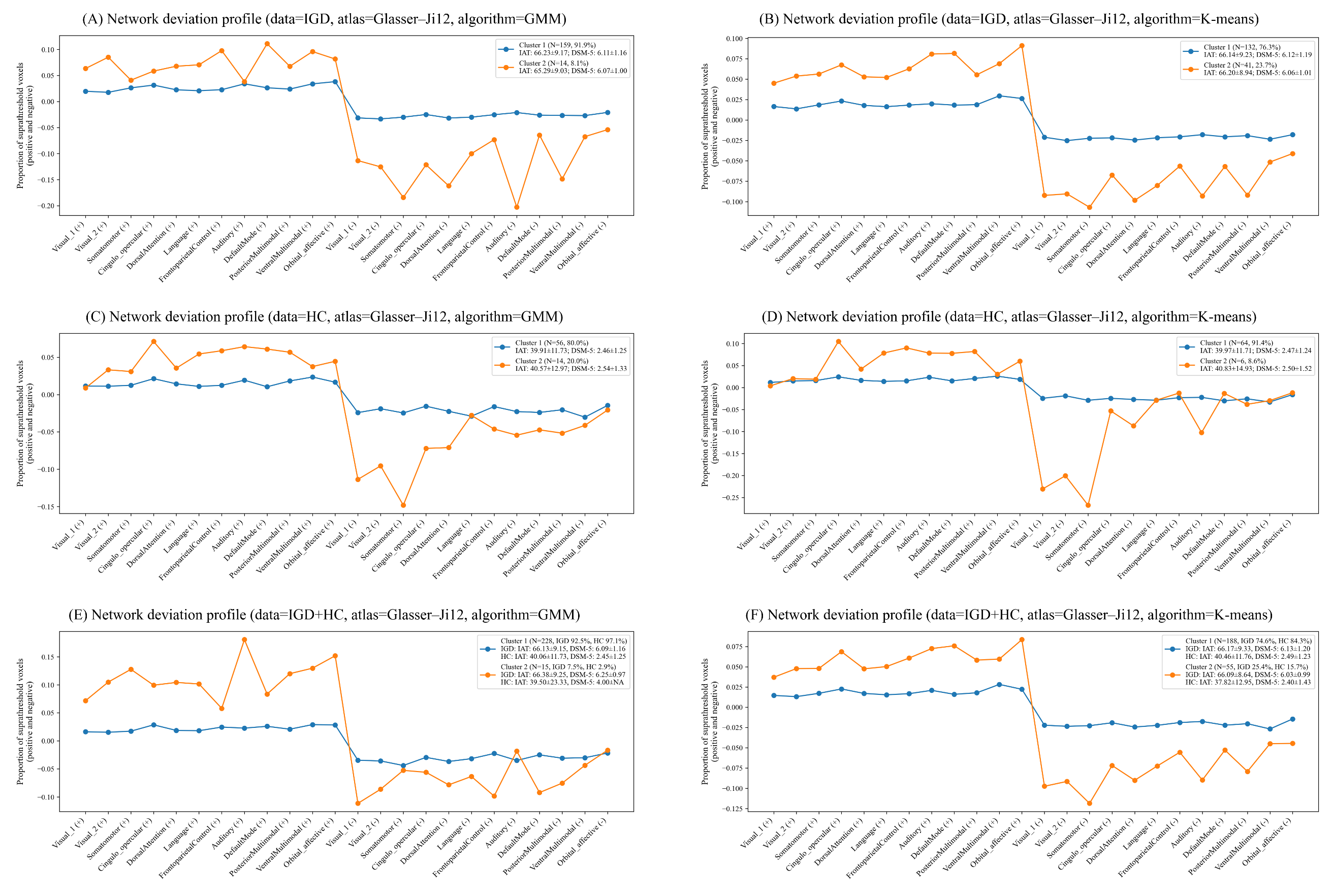
**

**Figure S9 Cluster-wise network deviation profiles under the Glasser-Ji12 atlas (three data × two algorithms)**

Notes: Three data: “IGD” —all the IGD participants data, “HC” —the out-of-sample 30% HC participants data, and “IGD+HC” —the combined IGD+out-of-sample 30% HC participants data; Two algorithms: GMM and K-means. The “(+)” and “(−)” symbols shown in the x-axis labels indicate positive deviations (Z > 2) and negative deviations (Z < −2), respectively. Accordingly, the proportion of suprathreshold positive deviations within each network is plotted as a positive value, whereas the proportion of suprathreshold negative deviations is plotted as the negative of that proportion (i.e., multiplied by −1). This sign convention is used purely for visualization, to clearly distinguish positive from negative deviations within a single profile. The legend reports, for each cluster, the cluster size and percentage, as well as the mean ± SD of IAT and DSM-5 scores (computed within the corresponding group when applicable). In particular, for the combined IGD+HC data, the IGD% and HC% shown are within-group percentages (i.e., relative to the total number of IGD or HC participants, respectively), rather than percentages of the total cluster size, to more clearly show how many individuals from each group (IGD vs. HC) were assigned to each cluster.

Abbreviations: IGD, Internet Gaming Disorder; HC, Healthy Control; IAT, Internet Addiction Test; DSM-5, the fifth edition of Diagnostic and Statistical Manual of Mental Disorders; GMM, Gaussian Mixture Modeling; Glasser-Ji12, 12-network parcellation derived from the Glasser 360-ROI atlas combined with the Ji 12-network partition.
